# Outcome of reminder text messages intervention on completion of routine immunization in rural areas, Nigeria

**DOI:** 10.1093/heapro/daaa092

**Published:** 2020-10-15

**Authors:** Oladimeji Oladepo, Isaac Oluwafemi Dipeolu, Opeyemi Oladunni

**Affiliations:** Department of Health Promotion and Education, Faculty of Public Health, Oladele Ajose Building, College of Medicine, UCH, Queen Elizabeth road, University of Ibadan, Ibadan, 200001, Nigeria

**Keywords:** vaccination, rural areas, text messaging, Nigeria

## Abstract

Completion of routine immunization for infants has been a challenge in Nigeria, and existing strategies implemented to promote immunization coverage yielded limited success. The use of reminder short services message (SMS) in mobilizing mothers of infants, especially in rural areas with lower immunization coverage has been suggested. This study investigated the effect of reminder SMS sent to mothers in rural communities on full and timely completion of routine childhood immunization. A quasi-experimental design was adopted, 3500 mothers of infants were categorized into the Intervention and Control groups recruited at various Primary Healthcare Centres in 6 states and the FCT, Nigeria. Reminder SMS were sent to mothers in the intervention group for 10 months. We adopted mixed methods of data collection, significance level set at *p* = 0.05. Majority of respondents were married (Control 94.3%; Intervention 95.5%), have experienced multiple births (Control 79.0%; Intervention 74.9%). Adherence to routine immunization appointment dates and completion of all immunizations was higher in the Intervention group (76.0%) compared with the Control (73.3%). A significant association between adherence to appointment dates and completeness of routine immunization vaccine was found. The Intervention group had a significantly higher completion rate for measles and yellow fever vaccines (55.3%; 75.9%) compared with the Control group (26.8%; 23.9%). Qualitative findings revealed positive comments from mothers in the intervention group that the messages increase awareness of immunization dates, assisted in readjusting their time which influenced timely completion. Interventions using reminder SMS enhanced infant immunization delivery; we recommend scale-up and integration into the health system to increase national immunization coverage.

## BACKGROUND

The Expanded Programme on Immunization (EPI), introduced in 1978 to provide routine immunization to children less than the age of 2 years, recorded initial but intermittent successes. The optimum level recorded in the early 1990s was 81.5%, but since the period of success, Nigeria has witnessed a consistent reduction in immunization coverage. By 1996, the national data showed <30% coverage for all antigens, and this decreased to 12.9% in 2003 (Babalola and Olabisi, 2004; Ophori *et al.*, 2014) which was among the lowest in the world and the worst in the West African sub-region. Two National Demographic Health Surveys conducted later showed a marginal increase in national childhood immunization in 2008 (22.7%) and 2013 (25. 4%). Of special concern is the state of measles vaccine coverage which has remained low (National Population Commission [Nigeria] and ICF International, 2014). Thus, national vaccination coverage has not exceeded 30% in the last three decades (Table 1). The NDHS 2013 report on the immunization coverage status in the six geo-political zones and the Federal Capital Territory (FCT) shows lower vaccination coverage in the northern zones (North West, 9.6%; North East, 14.2%; North Central, 26.9%) compared with the south (South West, 40.9%; South East, 51.7%; South South, 52.0%). The FCT recorded the highest coverage of 60.9%. The immunization coverage in urban and rural areas in the country was 42.5% and 15.8%, respectively.

**Table 1:** Percentage of children age 12 − 23 months who received specific vaccines at any time before the survey^a^

Source of information	BCG	DPT^b^			Polio^c^				Measles	All basic vaccinations^d^	No. vaccination	No. of children
		1	2	3	0	1	2	3				
2003 NDHS	48.3	42.6	31.7	21.4	27.8	67.2	52.3	29.4	35.9	12.9	26.5	999
2008 NDHS	49.7	52.0	44.7	35.4	36.7	67.8	57.2	38.7	41.4	22.7	28.7	4945
2013 NDHS	51.2	50.6	45.6	38.2	46.8	76.5	69.9	53.6	42.1	25.4	20.7	5900

*Source:*National Population Commission [Nigeria] and ICF International, 2014).

a According to vaccination card or the mother’s verbal report.

b Includes pentavalent.

c Polio 0 is the polio vaccination given at birth.

d BCG, measles and three doses each of DPT and polio vaccine (excluding polio vaccine given at birth).

Past efforts made by the Nigerian government to address the low childhood routine immunization coverage [adaption of WHO/Reaching Every District (RED)] approach in response to the decline or stagnation in routine immunization coverage (Vandelaer *et al.*, 2008; IMMUNIZATIONbasics [USAID], 2009) have yielded limited success (Abdulraheem *et al.*, 2011; Jegede and Owumi, 2013; Ataguba *et al.*, 2016). Other efforts include the Expanded Programme on Immunization (EPI) (Adedokun *et al.*, 2017), National Immunisation Days {NIDS}, Immunisation Plus Days {IPDs}—days set aside to strengthen the fight against polio in Nigeria (IMMUNIZATIONbasics [USAID], 2009) and public enlightenment; these have also yielded limited success (Abdulraheem *et al.*, 2011; Jegede and Owumi, 2013). In the light of this, there is an urgent need to test innovative approaches such as the use of reminder mobile phone text messages or Short Services Messages (SMS) in mobilizing mothers of infants especially in the rural areas with lower immunization coverage. Studies conducted in Pakistan, Guatemala and Zimbabwe concluded that SMS intervention improved routine vaccination coverage (Kazi *et al.*, 2018), evidence from the application of SMS technology in other countries show that it is feasible to implement in low- and middle-income countries (Domek *et al.*, 2016) and increase in immunization coverage was attributed to the use of SMS (Bangure *et al.*, 2015). Thus, such mHealth applications are useful because they capitalize on existing mobile phone infrastructure and an audience already familiar with the technology in addition to ease of use (Cole-Lewis and Kershaw, 2010; Marshall *et al.*, 2013), low cost and public interests (Terry, 2008).

However, evidence demonstrating the impact of reminder SMS as a tool for enhancing the uptake and timely completion of routine immunization in Nigeria remains scarce despite the evidence of mobile phone ownership in Nigeria (urban 88.6%; rural 64.8%) (National Population Commission [Nigeria] and ICF International, 2014). Thus, the goal of this study was to investigate the effect of reminder text messages sent to mothers of infants on full and timely completion of routine childhood immunization. Findings from this study would contribute to the body of literature, which is currently scarce and could be used to influence m-Health policy dialogue in Nigeria.

## METHODS

### Study setting

This study was quasi-experimental in design; two groups (Intervention and Control) were assessed at Baseline and Endline.

The study was conducted in Nigeria between November 2017 and May 2019. Six states (each state representing a geo-political zone in Nigeria) were selected through simple random sampling in addition to the FCT that was purposively selected. Two rural Local Government Areas (LGAs) from each state, including two area councils from the FCT, were randomly selected and assigned to the Intervention and Control groups.

Mothers of infants aged 0 − 2 months attending immunization clinics in the Primary Healthcare Centres (PHCs) in the 14 LGAs across the 6 states and the FCT and who owned at least 1 mobile phone during the period (study population) were randomly enrolled in the study. Specifically, mothers with infants between the age of 0 and 2 months were recruited because infants within this age range can still access immunization services till the 14th week of birth. After the 14th week, the next visit for routine immunization for such an infant is at the 9th month when the child is due for measles and yellow fever vaccination. Therefore, the period between the 14th week and 9th month (8 − 10 months, depending on the age of the infant when the mother was recruited) was used for the intervention. The sample size was calculated using the formulas adapted from Kirkwood and Sterne (2010) and Lwanga et al. (1991). A sample size of 166 eligible mothers of infants for each LGA was obtained but increased to 250 to adjust for attrition. This resulted in a minimum of a total sample size of 3500 mothers of infants for all 14 LGAs in all the 6 geo-political zones and the FCT.

### Intervention

The reminder text messages were based on focus group discussions (FGDs) with mothers at PHCs and supplemented with suggestions from questionnaires. The output from the data collection methods was used to design the content, language and time of delivery. The messages were first developed in English language and translated to Yoruba, Ijaw, Hausa and Igbo languages and focussed on the following: need to keep the next routine immunization appointments, benefits of keeping routine immunization appointments, benefits of timely and full completion of all basic routine immunizations, the consequences of refusal/non-completion of all basic routine immunizations on children’s health and locations of healthcare facilities with routine immunization services. The messages were customized and coded as *Riga kafi* for the Hausas, *Ogwu mgbochi* for the Ibos and *Abiye lomo* for the Yorubas.

These messages were sent to mobile phones of mothers of infants in the intervention group and their significant others (relatives named by mothers) for 10 months using a bulk SMS account created on a platform of one of bulk SMS service providers inclusive of the mothers’ phone numbers in the contact list. The messages were sent at scheduled intervals (three times a week between 7.00 and 7.15 am). At the end of the 5th month, Research Assistants visited the mothers’ residence to verify that their house addresses and telephone numbers were correct, in case either of the two had changed.

### Control group

Mothers of infants in the Control group received no routine immunization-related message. However, they were given flyers on the importance of adequate child nutrition and growth monitoring.

### Data collection

Routine immunization clinics or services are not available in all the Primary Health Care (PHC) centres in the rural LGAs. For this study, four PHCs where routine immunization clinics/services are rendered in an LGA were selected. A mixed-method of data collection was used. FGD sessions were conducted among consenting mothers of infants, and participants were selected from the list of mothers of infants who came to the clinic and registered their infants for immunization on the day of visit to the centres. A validated FGD guide was used to facilitate the discussion and collect data of infant mothers’ perceptions on immunization and use of reminder messages among mothers. Each FGD session consisted of eight consenting participants who indicated an interest in the discussion. In all, a total of 42 FGDs were conducted (3 FGD sessions per LGA). A Key Informant Interview (KII) guide was used to obtain information on opinions about the project from the health workers in the PHC facilities where the study was conducted. Overall, a total of 28 KIIs were conducted among healthcare workers who are involved in the immunization programme (2 healthcare workers per LGA). A validated semi-structured questionnaire was used to measure immunization knowledge, attitude, reported and actual vaccines received by infants from mothers. Also, an observational checklist was used to document all childhood routine immunizations from the index child immunization card including the date a child is expected to be immunized, the actual date such a child is vaccinated and the next routine immunization appointment for the child. Survey respondents were consenting mothers of infants selected from the list of those who registered their infants for immunization on the day of visit to the PHCs. Mothers of infants recruited for the survey did not participate in the FGD sessions.

### Ethical considerations

The study was approved by the University of Ibadan/University College Hospital Ethics Committee (UI/EC/17/0561). Written informed consent was obtained from respondents after explaining the objectives of the study, assurance of their privacy and confidentiality of information provided, and that participation is voluntary. During the intervention phase, mothers of infants in the Intervention group received immunization-related reminder text messages for about 10 months. Mothers of infants in the Control group did not receive any reminder text messages; however, they were provided with health information flyers on the importance of observing adequate child nutrition and growth monitoring.

No information on the benefits and importance of completing all routine immunization antigens for their children are withheld from any groups of mothers by this study. Healthcare workers involved in routine immunization services usually give health talks on the benefits and importance of completion of all vaccine antigens for their children, and the danger of defaulting and incomplete vaccination. So, all mothers of infants who come to the clinic receive these health talks regularly before their infants are vaccinated. This is done routinely across all states in Nigeria on routine immunization clinic days. Also, most mothers of infants are exposed to other routine immunization-related messages received on media, either radio or television. The reminder SMS among the Intervention group complemented the usual clinic health talks and information given to every mother who attends immunization clinics irrespective of their states or locations.

### Analysis

Qualitative information was transcribed from voice recorders and handwritten notes, word-processed and edited, entered into the computer and subjected to content analysis using the NVivo software. Quantitative data were entered into a computer using IBM/SPSS (version 21.0) and analysis presented in descriptive and inferential statistics using *t*-Test, Chi-square and Fisher’s exact tests with statistical significance set at *p* = 0.05.

The hypotheses tested in the study were: there is no significant association between the experimental and control groups in respect to (i) adherence with appointment dates and completion rate of immunization for children aged 0 − 11 months and (ii) status and completion rate of immunization for children aged 0 − 11 months.

## RESULTS

### Demographics

Majority of the mothers in the Control—94.3%; Intervention—95.5% groups were married, and most were Christians (Control—59.0%; Intervention—60.7%). About half of the respondents (Control—45.8%; Intervention—53.2%) had a secondary school education, and above, and trading was the most prominent occupation (40.1% and 34.2% in Control and Intervention groups, respectively). The mean age of respondents was 27.98 ± 5.2 and 27.52 ± 5.3 in Control and Intervention groups, and the majority had multiple births (Control—79%; Intervention—74.9%). Health workers were the primary source of information (Control—55.4%; Intervention—67.5%). The respondent’s average monthly income was 9372.07 for the control group and 12 048.19 for the intervention group (Table 2).

**Table 2:** Respondents’ socio-demographic characteristics (*N* = 3440)

Demographic characteristics	Control		Intervention	
	No.	%	No.	%
Marital status				
Married	1499	94.3	1479	95.5
Single	25	1.6	24	1.5
Others^a^	66	4.2	46	5.7
Religion				
Christianity	939	59.0	941	60.7
Islam	584	36.7	570	36.8
Traditional	7	0.4	0	0.0
No response	61	3.8	38	2.5
Highest education qualification				
No formal education	118	7.4	94	6.1
Primary school not completed	130	8.2	74	4.8
Primary school completed	267	16.8	154	9.9
Arabic/Quranic	137	8.6	110	7.1
Secondary school not completed	136	8.6	212	13.7
Secondary school completed	488	30.7	604	39.0
NCE	156	9.8	146	9.5
Bachelor/HND	75	4.7	57	3.7
Postgraduate	9	0.6	15	1.0
No response	74	4.7	83	5.4
Main occupation				
Trading	637	40.1	530	34.2
Artisan	133	8.4	113	7.3
Civil/public servant	120	7.6	111	7.1
Maid	1	0.1	0	0.0
House wife	156	9.8	401	25.9
Farmer	144	9.1	141	9.1
Professional^b^	4	0.2	7	0.5
Student	11	0.7	34	2.2
Self-employed	5	0.3	2	0.1
Unemployed	368	23.7	189	12.2
Ethnic group				
Yoruba	187	11.8	216	13.9
Igbo	321	20.2	300	19.4
Hausa	494	31.1	477	30.8
Others	585	36.7	556	35.9
Age (as at the last birthday)				
<20 years	63	4.4	67	4.5
20 − 29 years	831	58.6	928	62.5
30 − 39 years	482	34.0	456	30.7
>40 years	42	2.9	34	2.3
Mean (SD)	27.98 ± 5.2		27.52 ±5.3	
Birth experience				
First child	321	21.0	374	25.1
Multiple births	1204	79.0	1118	74.9
Source of information on immunization^c^				
Health worker/health facility	881	55.4	1045	67.5
Public announcement/town crier	427	29.1	289	18.7
Church or mosque	188	11.8	61	3.9
Media	795	50.0	1310	84.6
Friend or neighbour or spouse	96	6.0	130	8.4
School	4	0.3	3	0.2
Self	5	0.3	12	0.8
Others	2379	149.9	2016	130.2
Average monthly income from all sources				
<1000	117	16.5	77	11.5
1000–9999	338	47.6	229	34.2
10 000–19 999	146	20.6	223	33.3
20 000–29 999	55	7.7	67	10.0
30 000–39 999	28	3.9	29	4.3
40 000–49 999	9	1.3	12	1.8
>50 000	17	2.4	20	3.0
Mean	9372.07		12 048.19	

a Others include the divorced, widowed, co-habiting and the non-response.

b Professional include lawyer, pharmacist, nurse and health worker.

c Multiple responses.

### Mothers’ overall compliance with routine immunization appointment dates and completion of all immunization

**Fig. 1:**
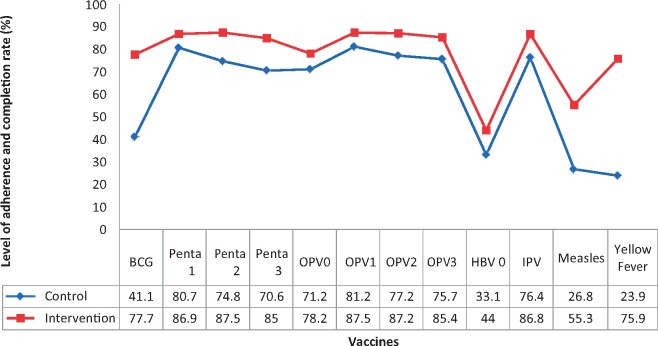
Respondents’ level of adherence to immunization appointment dates and completion rate.

The overall level of adherence to routine immunization appointment date and completion of all immunization was significantly greater in the intervention group compared with the Control (76.0 versus 73.3%, *p* = 0.00) (Table 3). Exploration of the outcome of the intervention showed a significant difference between the two groups in the completion of each vaccine at different levels. For BCG, the completion rate was 41.1% in the Control group while in the Intervention group, the completion rate was 77.7%. More importantly, respondents in the Intervention group had a significantly high completion rate in measles (55.3%) and yellow fever (75.9%) vaccines that are obtained at the 9th month when compared with the Control group (Figure 1).

**Table 3:** Overall mothers’ level of adherence with appointment dates and completion rate by different routine immunization vaccines

Variable	Adherence with the appointment dates and completion rate						Chi-square test		
	Control		Intervention		Total		χ^2^	df	*p*-Value
	No.	%	No.	%	No.	%			
BCG	490	41.1	951	77.7	1441	59.6	97.3	6	0.000
Pentavalent 1	938	80.7	1082	86.9	2020	83.9	313.5	6	0.000
Pentavalent 2	856	74.8	1076	87.5	1932	81.3	340.7	6	0.000
Pentavalent 3	799	70.6	1035	85.0	1834	78.0	364.1	6	0.000
Oral polio vaccine 0	805	71.2	945	78.2	1750	74.8	296.5	6	0.000
Oral polio vaccine 1	941	81.2	1075	87.5	2016	84.4	308. 0	6	0.000
Oral polio vaccine 2	886	77.2	1055	87.2	1951	82.3	356.6	6	0.000
Oral polio vaccine 3	855	75.7	1032	85.4	1887	80.7	403.1	6	0.000
HBV 0	340	33.1	492	44.0	832	38.8	171.6	6	0.000
IPV	827	76.4	1022	86.8	1849	81.8	612.2	6	0.000
Measles	289	26.8	637	55.3	926	41.5	235.9	6	0.000
Yellow fever	115	23.9	192	75.9	307	41.8	246.4	6	0.000

Further breakdown by states (Table 4) indicates differences in the overall completion rate exist across Intervention and Control LGAs in the selected states. Intervention groups in Abia, Benue, Katsina and Ondo states had significantly higher vaccination completion rates compared with the control group. Still, a reversal was noted in two states (Bauchi and Bayelsa states) where the Control significantly performed better. The FCT showed no significant difference in immunization completion rates.

**Table 4:** State comparison of immunization completion rate

State	LGA (group)	Completion rate (%)	Fisher’s exact test	*p*-Value
Abia	Osisioma Ngwa (intervention)	65.8	24.421	0.000
	Bende	41.5		
Bauchi	Kirfi (intervention)	62.7	62.540	0.000
	Ganjuwa	92.7		
Bayelsa	Kolokuma/Opokuma (intervention)	64.8	2.108	0.147
	Ogbia	71.9		
Benue	Otukpo (intervention)	78.4	5.291	0.021
	Gboko	68.8		
FCT	Kwali (intervention)	85.6	18.685	0.000
	Bwari	96.7		
Katsina	Malumfashi (intervention)	79.7	37.504	0.000
	Jibia	52.6		
Ondo	Idanre (intervention)	92.6	12.966	0.000
	Akure North	81.5		

The qualitative data on completion/timely appointment support the quantitative results with positive comments of mothers in the intervention group about the reminder messages increasing their full and timely completion of routine immunization of their children. One mother said,

I receive text messages on my phone every week at least twice. I like the messages; it has really helped me to complete my immunisation schedule.

(Mother A, from one of the South East LGAs)

Another mother noted:

I was very impressed because it prepares me against (next) immunisation and also made me to know, to get awareness that this immunisation is tomorrow for me to adjust my time if I should have any other thing to do, do it on time. (Mother B, from one of the FCT Area Councils)

Another mother stated:

Immediately after the message, I crosscheck my child’s immunisation card to be sure of the date.

(Mother C, from one of the North Central LGAs)

Furthermore, the expressed satisfaction by the frontline health workers in PHCs in the intervention LGAS lend credence to the quantitative data. One of them remarked,

Ah ah, yes I have so many expectations from the programme, I have so many even with that SMS it has increased the number of mothers coming for immunisation and completion. It’s really a good programme we have been yearning for it and still yearning for the continuity of the programme. Our people really appreciate it. One of my expectations also is that people should come out for the immunisation programme, they really came out. They are even happy; they showed us the text messages.

(Nurse A, from one of the South West LGAs)

Another one concluded:

It helps the health workers in meeting their targets … helps to track mothers.

(A Community Health Extension Worker [CHEW] from one of North Central LGAs)

## DISCUSSION

Overall, the timely completion of routine immunization by Intervention group is significantly higher than the Control group, which can be attributable to the reminder messages intervention. Similar results have been reported in Pakistan in which 76% versus 71.3% of Intervention and Control groups, respectively, completed required vaccination at the end 14th week of the study (Kazi *et al.*, 2018). Another study demonstrated that 97.0% in the intervention group and 82.0% in the non-intervention group completed immunizations at 6 weeks, and 95% and 75%, respectively, at 14 weeks (Bangure *et al.*, 2015). Of special note in this study is the increased rates of appointment keeping and completion among infants in the intervention group (55.3% for measles and 75.9% for yellow fever). The immunization coverage in previous national surveys for measles in 2003, 2008 and 2013 were 35.9%, 41.4% and 42.1%, respectively.

The finding which showed that the Control group had significantly higher coverage than the Intervention group in Bauchi and Bayelsa states was unexpected. However, reports from the health facilities where the study was conducted revealed that there were some ongoing parallel interventions in Bauchi state. The first was the Maternal and Child Health component of the Subsidy Reinvestment and Empowerment Programme (SURE-P) in Nigeria, which aspires to contribute to the reduction of maternal mortality and new-born morbidity. The other was a WHO-supported programme known as Routine Immunization Intensification. At the time the study was conducted, we are unaware of parallel interventions going on in the two states, which makes it more likely that the results could be attributed to the intervention. Also, the Intervention LGA in Bayelsa state experienced flooding during the period that led to the displacement of some participants, thereby affecting the compliance rate. This incident posed a challenge to the present study.

This study has some strengths: first, reminder text messages were sent timely according to the study protocol. Second, by enrolling mothers of infants at health facilities, selection bias was minimized compared with what would have been if they were enrolled in their various homes; bearing in mind that routine immunization can only be given at health facilities irrespective of where a woman delivered her baby. The positive comments of mothers in the intervention group about the SMS messages increasing their awareness of immunization dates, assisting them in readjusting their time, influencing their completion of the immunizations and advocating for the continuation of this programme lend credence to the value attached to this innovation.

There were some limitations to the present study. First, the authors could not monitor the delivery status of the SMS messages as it was unidirectional. It will be desirable in future studies that a link of the delivery status of sent SMS messages should be established to monitor actual delivery to individual receiver. Second, 60 questionnaires were inadequately completed (1.7%), but this did not compromise the quality of data collected as the minimum sample for the study was obtained in all the LGAs.

## CONCLUSION

This study shows that sending one-way reminder text SMS messages to mothers of infants with phone ownership promotes higher full and timely completion of routine childhood immunization. The study thus contributes to the evidence base on the effectiveness and acceptance of health text messaging for improved childhood immunization and variation in different settings. Findings also provided trusted evidence for decision making for child immunization health outcomes and services and contributed to the body of literature, which is currently scarce. Finally, it has provided a better understanding of the mechanisms through which mobile phone technology supports community-based health care delivery.

## RECOMMENDATIONS

Based on the findings of this study, the following are recommended for the following agencies of government—Federal Ministry of Health, the National Primary Healthcare Development Agency as well as all States Primary Healthcare Development Agencies:

1. Scaling-up this project to build a national body of evidence with wider geographic spread to other states in Nigeria for increasing national immunization coverage.
2. Exploring reminder text messaging as a viable channel for health education to complement conventional media messages. This would serve as a reinforcement of the media messages.
3. Funding should be provided for developing, producing and distributing training materials manuals/guidelines to researchers, PHC coordinators and maternal and child health focal persons to guide the replication of implementation of this project. And with adequate support from researchers as part of their technical contribution in collaboration with communication experts.
4. Building capacity of frontline health workers nationwide on the use of this innovation, and integrating routine immunization data generated into a national database.
5. Integrating m-Health strategy by policymakers into the existing national immunization policy.

## AVAILABILITY OF DATA AND MATERIALS

Data and materials for the study are available upon request.

## ETHICAL APPROVAL

Ethical approval for the study was obtained from the UI/UCH Ethics Review Committee, College of Medicine, University of Ibadan, Nigeria (UI/EC/17/0561). We obtain written informed consent from all the respondents, they are competent, have the liberty and agency to make an informed decision. Parental or legal guardian consent to recruit respondents who are 14 years old into the study was not necessary; the majority of the respondents are married, wives, mothers and are in their husbands’ house. ‘Under-aged’ mothers are largely based in the Northern part of Nigeria; the dominant religion in this region (Islam) and the Sharia Law allow teenagers as young as 14 years old girls to be married and become independent of their parents on decisions affecting their families including their children.
